# Effects of Altitude, Slope Aspect, and Soil Depth on Soil Properties and Herbaceous Root Distribution in Honghe Hani Rice Terraces

**DOI:** 10.3390/plants15142144

**Published:** 2026-07-11

**Authors:** Linlin Huang, Xuesen Zhang, Xin Wang, Ruihong Wang, Ruizhang Wang, Xiaoqin Zhang, Yingdu Sun

**Affiliations:** 1 College of Civil Engineering and Architecture, China Three Gorges University, Yichang 443002, China; 2 Water Conservancy and Hydropower Engineering Geological Investigation Consultation and Planning Institute in Honghe Hani and Yi Autonomous Prefecture, Mengzi 661199, China

**Keywords:** terrace systems, soil physicochemical properties, root distribution, elevation gradient, spatial heterogeneity

## Abstract

To elucidate the elevational differentiation patterns of soil physical properties and nutrient contents within the Yuanyang Hani Terrace system and to clarify their influence on the spatial distribution of vegetation root systems, we systematically collected soil and root samples across varying elevations, slope aspects, and soil depths. A combination of soil physicochemical analyses, root distribution quantification, correlation analysis, and principal component analysis (PCA) was employed. The results indicated that soil bulk density exhibited a unimodal trend along the elevation gradient, initially decreasing and then increasing, with the following order: high-elevation sunny slope > high-elevation shady slope > low-elevation sunny slope > low-elevation shady slope > mid-elevation sunny slope > mid-elevation shady slope. The lowest bulk density values (1.10–1.17 g cm^−3^) were observed at mid-elevations, where porosity and infiltration rates reached their maxima (>54% and 1.31–1.34 mm min^−1^, respectively), whereas the poorest conditions were found at high elevations. Shady slopes consistently outperformed sunny slopes, with the greatest aspect-induced divergence occurring at mid-elevations (bulk density difference: 0.079 g cm^−3^). An anomalous combination of a sharp increase in bulk density (1.33 g cm^−3^), alongside simultaneous peak porosity (54.74%) and infiltration rate (1.32 mm min^−1^), was detected in the 20–40 cm layer, which was attributed to compaction-induced alterations in pore configuration while preserving preferential flow pathways. Most nutrient variables exhibited optimal levels at mid-elevations; however, total potassium and available phosphorus displayed maximal values at low elevations, likely related to chemical weathering intensity and plant uptake competition. Root systems were predominantly concentrated in the surface layer (0–20 cm), accounting for 87.88% of total root abundance, and showed significant positive correlations with soil moisture and porosity (r ≥ 0.89) and significant negative correlations with bulk density (r ≤ −0.97). The first principal component of PCA (explaining 73.4% of total variance) was dominated by moisture, porosity, and infiltration rate, whereas nutrient loadings were relatively low, indicating that physical hydrological processes constituted the primary drivers of environmental differentiation. Collectively, these findings elucidate the coordinated regulation of soil multi-attribute variations by elevation, slope aspect, and soil depth, thereby providing a theoretical basis for the sustainable management of terrace agroecosystems.

## 1. Introduction

The Honghe Hani Rice Terraces, located in southern Yunnan Province, China, represent an agricultural heritage system developed over more than 1300 years by the Hani, Yi, and other ethnic groups. Recognized successively in 2010, 2013, and 2025 as a Globally Important Agricultural Heritage System (GIAHS), a UNESCO World Heritage Site, and a World Heritage Irrigation Structure, they stand as the only farming civilization model worldwide to hold these three prestigious designations simultaneously. The core feature of the terraces is a vertically structured “forest–village–terrace–water system” ecosystem. This system supports sustainable subtropical mountain agriculture over millennia through efficient water regulation and material cycling [1,2,3]. Driven by key environmental gradients such as elevation and slope aspect, the spatial redistribution of light, heat, and water profoundly influences soil physical, chemical, and biological processes in the terraces [4,5,6]. The coupling relationships among soil moisture, nutrients, and plant roots—as the most dynamic components—form the basis for maintaining terrace productivity, ecological stability, and cultural heritage values [7,8,9]. Water acts as the medium for nutrient transport and plant uptake [10,11,12]; nutrients provide the material foundation for system productivity [13,14]; and roots perform key functions, including water and nutrient absorption, carbon and nitrogen fixation, and soil structure maintenance [15,16,17]. Along elevational gradients, variations in temperature, precipitation, evaporation, and irrigation management lead to systematic differences in soil hydrothermal conditions, organic matter decomposition, nutrient availability, and the ecological strategies of crop and associated herbaceous plant roots [18,19,20,21].

In recent years, significant progress has been made in understanding the hydrological processes and soil fertility of the Hani Terrace ecosystem [22,23,24,25,26]. Studies confirm its remarkable “sponge” function, with the forest water-conservation zone, the terraces themselves, and the canal network constituting an efficient water regulation system [27]. Stable isotope tracing (δ^2^H, δ^18^O) indicates that atmospheric precipitation is the primary water source, with the forest soil layer playing a critical role in water storage and regulation [28,29,30]. Along the elevation gradient, soil water content shows a distinct spatial pattern [31], while soil organic carbon and total nitrogen generally increase with elevation [32,33]. In contrast, phosphorus availability, significantly influenced by pH and iron–aluminum oxides, exhibits an unclear elevational pattern [34,35]. Most existing studies have treated water and nutrients as relatively independent units, leaving the coordinated variation of “water–nutrient coupling” and its driving mechanisms along elevational gradients poorly understood. In particular, there is a lack of mechanistic understanding of how water regulates the spatial availability of nutrients [36,37,38]. Furthermore, how traditional management practices such as irrigation and fertilization interact with natural elevational gradients to shape water–nutrient patterns requires further investigation.

As the critical interface connecting plants and soil, root functional traits are essential for maintaining system stability [39,40,41]. Under alternating wet–dry cycles and micro-scale soil environments, rice roots adapt to water stress by adjusting architectural traits such as rooting depth and root length density. However, low temperatures at higher elevations may limit vertical root extension, leading to a more superficial distribution [42,43,44]. Systematic studies on rice and terrace-bank herbaceous plant roots across different elevations remain scarce [45], and current knowledge is largely focused on aboveground components, leaving the variation in root functional traits along elevational gradients and their ecological significance poorly explored. In particular, the role of herbaceous plant roots on terrace banks in maintaining system stability has been overlooked, resulting in a significant gap in understanding their ecological strategies [46]. Most current studies fail to effectively integrate root traits with soil environmental factors, making it difficult to reveal how belowground plant parts respond and adapt to environmental changes. Given that water and nutrients are key resources directly acquired by roots, complex feedback relationships exist between their availability and root morphology and growth strategies. Plants often adapt to heterogeneous resource distribution through morphological plasticity, such as adjustments in root–shoot ratio and increased fine root proportion [47,48]. Within the Hani Terrace agricultural heritage system, the interactions among water, nutrients, and roots remain largely hypothetical, lacking rigorous empirical support. While the elevation gradient provides an ideal natural platform for testing relevant ecological hypotheses, current research tends to emphasize how the soil environment shapes root traits, generally neglecting the feedback regulation of roots on water and nutrient cycles through processes such as resource uptake. Future studies should, within a bidirectional interaction framework, systematically investigate the coordinated evolution of water, nutrients, and roots to better reveal the mechanisms underlying the stability of the Hani Terrace ecosystem.

In summary, the current research exhibits the following core limitations: there is a lack of holistic and coupled analysis treating the “soil moisture–soil nutrients–plant roots” as a co-evolving system along altitude gradients; the mechanisms of how water and nutrient conditions drive the ecological adaptation strategies of root morphology, architecture, and functional traits, as well as how roots in turn regulate rhizosphere water and nutrient processes, remain unclear; furthermore, the role of herbaceous plant roots on terrace bunds in system material cycling and stability maintenance has been overlooked, and their interaction mechanisms with water and nutrient factors await clarification. These knowledge gaps hinder the prediction of how the water–nutrient–root interactions in terrace systems will respond under future climate change and management shifts, thereby constraining the development of adaptive management strategies. To address these issues, this study aims to systematically analyze the interaction mechanisms between soil moisture–nutrients and herbaceous plant roots on terrace bunds across different altitudes and slope aspects in the Honghe Hani Terraces and to reveal the ecological adaptation patterns along environmental gradients. The findings are expected to provide theoretical support for the conservation and sustainable management of this traditional agroecosystem.

## 2. Results

### 2.1. Distribution of Selected Soil Physical Properties Across Elevation Gradients

In the terrace ecosystem, soil physical properties exhibited significant differentiation with elevation, slope aspect, and soil depth. To eliminate the confounding effects of soil type variation, this study focused on the 0–100 cm depth interval, which represents the primary zone of root activity and is directly influenced by near-surface atmospheric processes. The soils in the study area are characterized as silty sand with a uniform texture and high representativeness, thereby justifying the analytical emphasis on this layer. The spatial differentiation of soil bulk density is jointly influenced by altitude and slope aspect. Along the elevational gradient, bulk density exhibits a V-shaped trend, initially decreasing and then increasing with altitude. The lowest values were observed at mid-elevation (1.17 ± 0.08 g cm^−3^ on the sunny slope and 1.10 ± 0.09 g cm^−3^ on the shady slope), whereas the highest values occurred at high elevation (1.35 ± 0.06 g cm^−3^ and 1.31 ± 0.05 g cm^−3^, respectively). At low elevation, intermediate values were recorded (1.28 ± 0.06 and 1.24 ± 0.07 g cm^−3^). Within the same elevational zone, bulk density on sunny slopes consistently exceeded that on shady slopes, with the difference increasing from 0.04 g cm^−3^ at low elevation to 0.04–0.05 g cm^−3^ at high elevation, indicating an intensification of slope aspect effects with increasing altitude. The elevated bulk density at low elevations is attributable to mechanical compaction from frequent agricultural activities and rapid organic matter mineralization. The marked reduction in bulk density at mid-elevation reflects well-developed soil aggregates and enhanced porosity in this zone. At high elevations, low temperature and high moisture conditions promote physical weathering while reducing bioturbation, leading to denser soil structures. Total porosity, natural water content, and steady infiltration rate exhibited trends opposite to those of bulk density. At low elevation, total porosity ranged from 49% to 51%, water content from 31% to 34%, and infiltration rate from 1.12 to 1.19 mm min^−1^. All three indicators reached their optimal levels at mid-elevation, with values of 54–55%, 39–43%, and 1.31–1.34 mm min^−1^, respectively. At high elevation, these parameters declined to their minimum values: porosity 46–47%, water content 25–29%, and infiltration rate 0.93–0.96 mm min^−1^. The loose and porous structure at mid-elevation significantly enhanced water retention and aeration capacity, thereby facilitating runoff regulation. Although high-elevation soils maintained relatively high moisture due to low temperatures, their low porosity and poor infiltration capacity impeded water movement, limiting the availability of effective water resources. With respect to slope aspect, shady slopes generally outperformed sunny slopes across all indicators; however, the infiltration rate on the shady slope at high elevation (0.93 mm min^−1^) was slightly lower than that on the sunny slope (0.96 mm min^−1^), a difference that may be related to subtle variations in surface soil microstructure, warranting further investigation. Overall, the altitudinal differentiation of soil physical properties governs the variation in ecohydrological functions. Mid-elevation terraced fields demonstrated pronounced advantages in soil structure and water regulation, whereas high- and low-elevation zones were constrained by soil compaction and anthropogenic disturbance, respectively, necessitating targeted management interventions. Soil porosity, water content, and infiltration rate varied strictly inversely with bulk density, consistently following the order mid-elevation > low elevation > high elevation. Among all combinations, the shady slope at mid-elevation exhibited the highest total porosity (54.63 ± 3.45%), water content (43.17 ± 3.87%), and steady infiltration rate (1.34 ± 0.02 mm min^−1^), whereas the corresponding values on the sunny slope were 54.43%, 38.98%, and 1.31 mm min^−1^. At low elevation, porosity on the shady and sunny slopes was 51.11% and 49.38%, water content 34.28% and 31.20%, and infiltration rates 1.19 and 1.12 mm min^−1^, respectively, representing substantial declines relative to mid-elevation. Although absolute water content at high elevation remained moderately high (29.06% on shady slopes and 25.42% on sunny slopes), the lowest porosity (47.16% and 46.33%, respectively) and dense structure resulted in sharply reduced infiltration rates of 0.93 and 0.96 mm min^−1^, the lowest among all sites. This suggests that water storage at high elevations depends more on reduced evaporation under low temperatures than on intrinsic pore structures favorable for water retention, implying poor accessibility of effective water resources.

**Figure 1:**
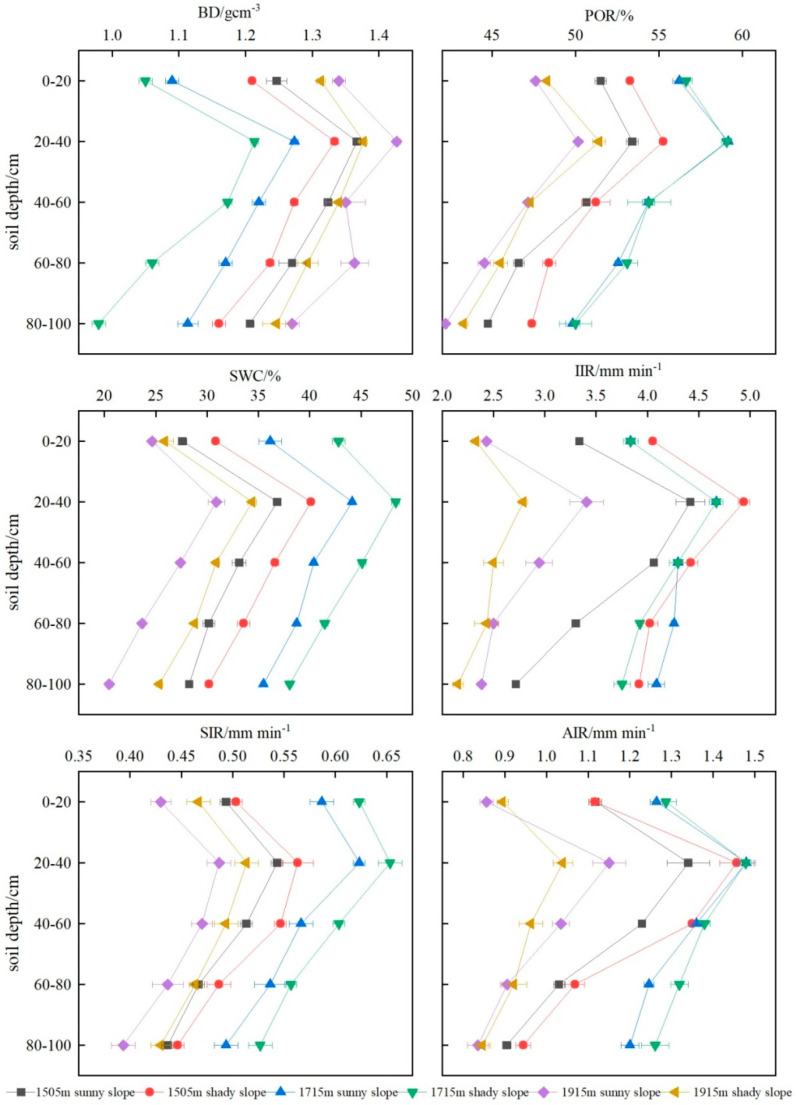
Distribution characteristics of basic soil physical properties across different altitudes.

Vertically, the 0–20 cm surface layer exhibited relatively low bulk density (1.21 g cm^−3^) and high porosity (52.25%) due to tillage disturbance and organic matter enrichment; however, its infiltration rate (1.09 mm min^−1^) and water content (31.33%) were lower than those in the 20–40 cm layer. In the >40 cm layer, bulk density decreased to its minimum (1.23 g cm^−3^), whereas porosity (48.51%) and water content (32.65%) also declined, with infiltration rate (1.10 mm min^−1^) at an intermediate level. This seemingly paradoxical result reveals the underlying mechanism of soil structural differentiation induced by repeated mechanical wheel traffic. Under compressive loading, macropores collapsed and were transformed into mesoporous matrix pores, thereby increasing bulk density at the macroscopic scale. Nevertheless, residual root channels and structural fissures did not close completely; instead, they formed a connected preferential flow network within the compacted layer. The interplay of these two effects resulted in enhanced water conductivity in this horizon compared with the surface layer, despite higher bulk density, indicating a non-typical hydraulic behavior characterized by compactness without concomitant impedance to water flow (Figure 1).

### 2.2. Distribution Characteristics of Soil Nutrients Along Altitudinal Gradients

**Figure 2:**
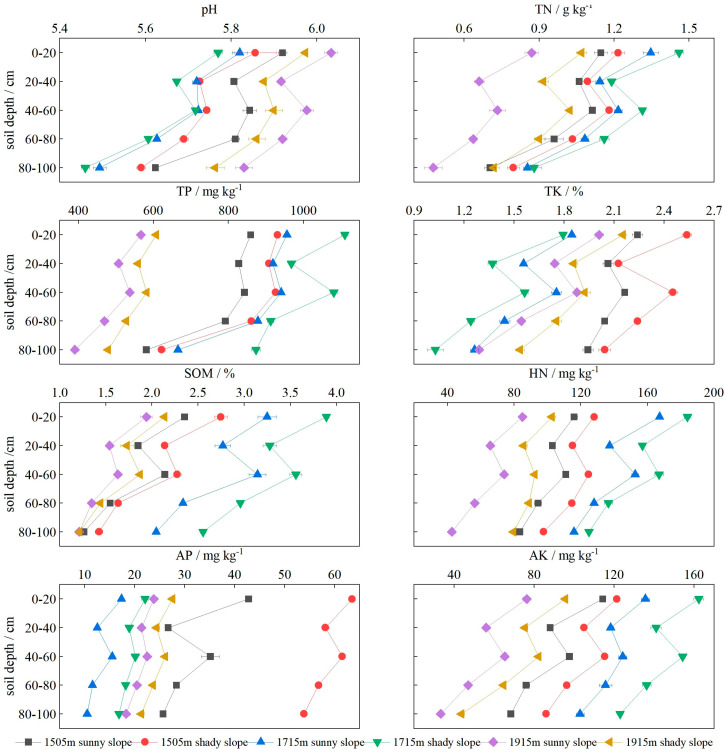
Distribution characteristics of soil nutrients across different elevations.

In terrace agroecosystems, soil pH, organic matter, and major nutrients—including total nitrogen, total potassium, total phosphorus, hydrolyzable nitrogen, available potassium, and available phosphorus—exhibit pronounced differentiation along vertical profiles, elevational gradients, and slope aspects. Vertically, all indicators display surface accumulation, with values generally decreasing, then increasing, and subsequently decreasing again with increasing depth. The surface layer (0–20 cm) contained the highest nutrient concentrations, with pH and organic matter at 5.90 ± 0.01 and 2.72 ± 0.67%, respectively, and peak values of total nitrogen (1.18 ± 0.22 g kg^−1^), total potassium (2.10 ± 0.25%), total phosphorus (837.87 ± 211.43 mg kg^−1^), hydrolyzable nitrogen (130.47 ± 37.88 mg kg^−1^), available potassium (100.89 ± 58.28 mg kg^−1^), and available phosphorus (32.86 ± 17.33 mg kg^−1^). Based on organic carbon content (converted from organic matter using a factor of 58%) and total nitrogen, the calculated C/N ratio in the surface soil was approximately 13.3, falling within the range of 8–15 commonly observed in subtropical mountain soils of China, which is consistent with typical carbon–nitrogen stoichiometric characteristics in this region. In the 20–40 cm layer, nutrient levels declined markedly; a recovery was observed in the 40–60 cm layer, though concentrations remained lower than those in the surface layer; below 60 cm, values continued to decrease down to 100 cm depth. The overall vertical sequence followed the order 0–20 cm > 40–60 cm > 20–40 cm > 60–80 cm > 80–100 cm, with surface layer concentrations being 1.4- to 1.7-fold greater than those in the deepest layer. This distribution is co-driven by pedogenic processes, water movement, and biological cycling: surface enrichment results from litter input, root exudation, and intense microbial activity; the 20–40 cm layer acts as an eluviation horizon, where soluble nutrients are lost with percolating water; in the 40–60 cm layer, adsorption of phosphorus and potassium by clay minerals and iron–aluminum oxides is enhanced, forming a weak illuviation horizon; below 60 cm, the parent material layer exhibits weak biological activity, poor aeration, and low mineralization rates, rendering it the most nutrient-depleted zone (Figure 2).

Along the elevational gradient, all nutrients except total potassium and available phosphorus were significantly higher at mid-elevation than at both low and high elevations (*p* < 0.05). Organic matter at mid-elevation (2.98 ± 0.56%) was approximately 1.54-fold and 1.85-fold greater than that at low (1.94 ± 0.47%) and high (1.61 ± 0.31%) elevations, respectively; total nitrogen, hydrolyzable nitrogen, and available potassium followed the same order. These differences are attributable to the optimal hydrothermal regime and the highest vegetation cover at mid-elevation, which favor both organic matter input and moderate mineralization. At low elevations, higher temperatures accelerate decomposition and, combined with intensive agricultural activities, lead to enhanced nutrient consumption and loss; at high elevations, low temperatures suppress plant productivity, resulting in insufficient exogenous organic matter inputs and limited accumulation despite slow decomposition. In contrast, total potassium and available phosphorus followed the order low elevation > high elevation > mid-elevation, opposite to the pattern observed for the other nutrients. This reversed trend is primarily controlled by parent material weathering type: intense chemical weathering at low elevations promotes the release of potassium from K-bearing minerals such as feldspar and also increases available phosphorus through dissolution of primary phosphate minerals; at high elevations, physical freeze–thaw weathering predominates, resulting in intermediate potassium release; at mid-elevations, vigorous plant growth leads to substantial uptake of potassium and phosphorus, while concurrent clay fixation of potassium and adsorption of phosphorus by iron–aluminum oxides reduce their available fractions. It should be noted that the above patterns were derived from comparisons among three specific elevations; further validation of continuous trends would require denser sampling intervals along the elevational gradient.

Slope aspect also exerted a significant influence on nutrient distribution, with overall nutrient contents being higher on shady slopes than on sunny slopes. Mean organic matter content was 2.32 ± 0.82% on shady slopes versus 2.02 ± 0.64% on sunny slopes. At mid-elevation, organic matter on shaded and sunny slopes was 1.57- and 1.48-fold higher than that at low elevation on the same aspect and 1.92- and 1.77-fold higher than that at high elevation, respectively. Shady slopes, characterized by shorter sunshine duration and higher humidity, experience slower organic matter decomposition and thus greater accumulation; sunny slopes, with stronger evaporation and greater temperature fluctuations, exhibit faster mineralization and weaker accumulation capacity. This aspect-induced pattern was consistent across all elevational zones, indicating that local hydrothermal configurations exert a universal regulatory effect on nutrient cycling.

In summary, soil pH, organic matter, total nitrogen, total phosphorus, hydrolyzable nitrogen, available potassium, and available phosphorus in the Yuanyang Hani terrace soils differed significantly among elevations, slope aspects, and soil depths (*p* < 0.05). The vertical distributions exhibited surface enrichment with weak illuviation characteristics. Across the three elevations, most nutrients (with the exception of total potassium and available phosphorus) were optimal at mid-elevation, while the aspect effect remained stable, with shady slopes generally outperforming sunny slopes.

### 2.3. Distribution Characteristics of Herbaceous Plant Roots at Different Altitudes

**Figure 3:**
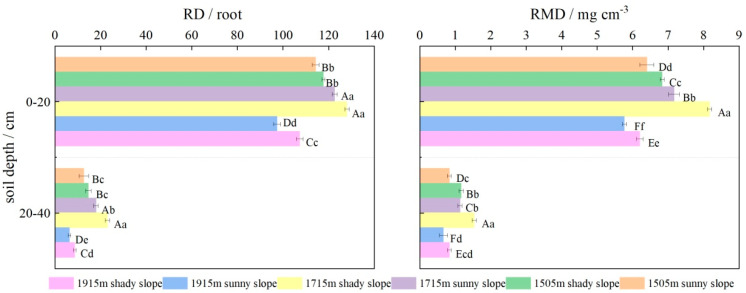
Root distribution showed pronounced surface aggregation. Uppercase letters indicate significant differences among elevations and aspects (*p* < 0.05), whereas lowercase letters indicate significant differences among soil layers (*p* < 0.05).

The vertical and spatial distribution characteristics of root systems in herbaceous plants in the Honghe region exhibit significant altitudinal gradients, slope aspect differences, and vertical soil heterogeneity. Systematic sampling analyses revealed that both root number and root mass followed a consistent pattern across altitudes, with the highest values observed at mid-altitude, followed by low-altitude, and the lowest at high-altitude, and were significantly greater on shady slopes than on sunny slopes. Furthermore, root biomass and morphological parameters in the 0–20 cm soil layer were significantly higher than those in the 20–40 cm layer, with shady slopes consistently outperforming sunny slopes within each depth interval. Specifically, the highest root number was recorded on the mid-altitude shaded slope (151 roots), followed by the mid-altitude sunny slope (141 roots), low-altitude shaded slope (133 roots), low-altitude sunny slope (127 roots), high-altitude shaded slope (116 roots), and high-altitude sunny slope (104 roots). The interaction between altitude and slope aspect was statistically significant (*p* < 0.05), with the ranking order being mid-altitude shaded > mid-altitude sunny > low-altitude shaded > low-altitude sunny > high-altitude shaded > high-altitude sunny. Within the same altitude, shady slopes exhibited root numbers of 4.20–11.90% higher than those on sunny slopes. This distribution pattern is primarily attributed to the optimal hydrothermal conditions and highest soil nutrient availability at mid-altitudes, whereas root development at high altitudes is constrained by low temperatures and at low altitudes by intensive anthropogenic disturbance and high surface evaporation (Figure 3).

In terms of vertical soil distribution, root number and root weight density in the 0–20 cm surface layer were significantly greater than those in the 20–40 cm layer. Across all samples, the mean root number in the 0–20 cm layer was 115 ± 11 roots, with the maximum root weight density observed on the mid-altitude shaded slope (8.17 ± 0.06 mg·cm^−3^). In contrast, the mean root number in the 20–40 cm layer decreased sharply to 14 ± 6 roots, representing a reduction of 87.88%. Within the same deeper layer, the highest root weight density still occurred on the mid-altitude shaded slope (1.53 ± 0.06 mg·cm^−3^), whereas the lowest values in both layers were found on the high-altitude sunny slope. Although significant differences were detected among most altitude–aspect combinations, no statistically significant difference was observed in root weight density between the low-altitude shaded slope and the high-altitude sunny slope in the 0–20 cm layer, suggesting that different limiting factors—anthropogenic disturbance at low altitude versus low temperature at high altitude—may lead to comparable root biomass accumulation. With increasing soil depth, soil compaction intensified while organic matter content and aeration declined, resulting in a marked reduction in root proliferative capacity, which collectively explains the pronounced surface aggregation of root systems.

### 2.4. Relationships Among Soil Moisture, Nutrients, and Root Systems

**Figure 4:**
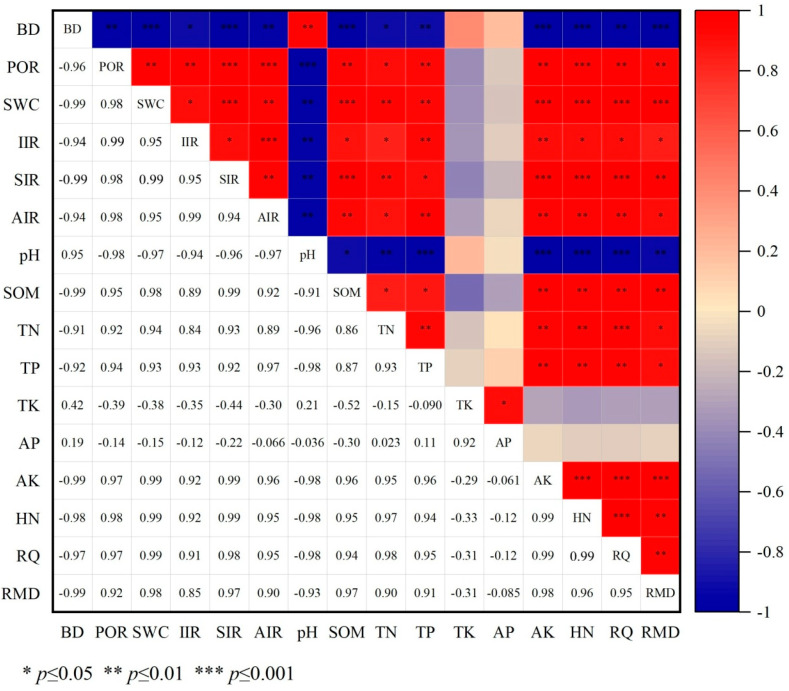
The correlation matrix among soil physicochemical properties, root traits, and topographical factors.

Based on the correlation matrix among soil physicochemical properties, root traits, and topographical factors (Figure 4), bulk density exhibited significant negative correlations with root number (r = −0.97) and root weight density (r = −0.99), indicating that increased soil compactness impeded root penetration and suppressed biomass accumulation. Porosity and soil water content were significantly positively correlated with all root parameters (r ≥ 0.92), with correlation coefficients of 0.99 and 0.98 against root number and root weight density, respectively. It should be noted, however, that these statistical relationships do not necessarily denote a unidirectional causal effect of moisture on root growth. In the study area, altitudinal and aspect gradients often covary with concomitant changes in hydrothermal regimes, leading to inherent spatial collinearity. Furthermore, in well-developed root systems, root turnover and subsequent decomposition may create biopores that increase soil macroporosity, thereby enhancing infiltration and water-holding capacity and resulting in elevated soil moisture. Hence, the observed strong correlations at least partly reflect the combined effects of reverse causality and confounding factors. Available potassium (r = 0.99 and 0.98) and hydrolyzable nitrogen (r = 0.99 and 0.96) were highly correlated with root traits, suggesting that these two labile nutrients exerted pronounced promoting effects on root development. Total potassium and available phosphorus did not show significant correlations with root parameters (r = −0.31 and −0.12, respectively), which may be attributable to the strongly alkaline soil pH in this region (pH correlated with root number and root mass at r = −0.98 and −0.93, respectively): under high-pH conditions, phosphorus is readily fixed by calcium, while potassium availability is constrained by adsorption onto clay minerals, rendering total contents inadequate proxies for actual nutrient supply. Organic matter, total nitrogen, and total phosphorus exhibited correlation coefficients ranging from 0.90 to 0.99 with root parameters, reflecting synergistic relationships between baseline soil fertility and root system development. Initial infiltration rate, steady infiltration rate, and average infiltration rate were all correlated with root number and root weight density at coefficients no less than 0.85, with steady infiltration rate achieving values of 0.98 and 0.97, respectively. Soils with higher infiltration capacity generally possess loose structures and continuous pore networks, which not only facilitate downward root penetration but also provide conduits for deep-layer water recharge, suggesting a positive feedback loop between physical habitat quality and root system expansion. Collectively, these findings indicate that elevation and slope aspect primarily influence the distributions of soil moisture, organic matter, total nutrients, and root biomass through coupled hydrothermal gradients and material transport processes.

**Figure 5:**
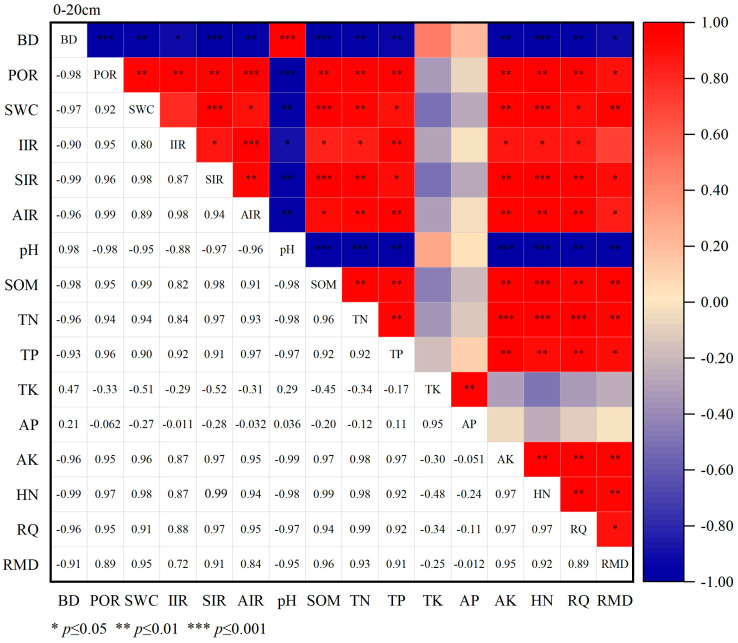
The correlation analysis among soil moisture, nutrients, and root traits across soil depths (0–20 cm).

**Figure 6:**
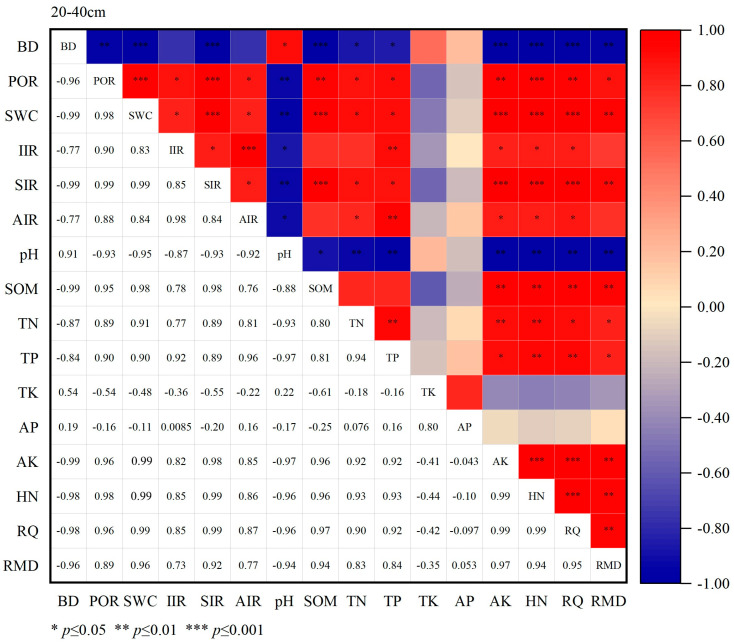
The correlation analysis among soil moisture, nutrients, and root traits across soil depths (20–40 cm).

**Figure 7:**
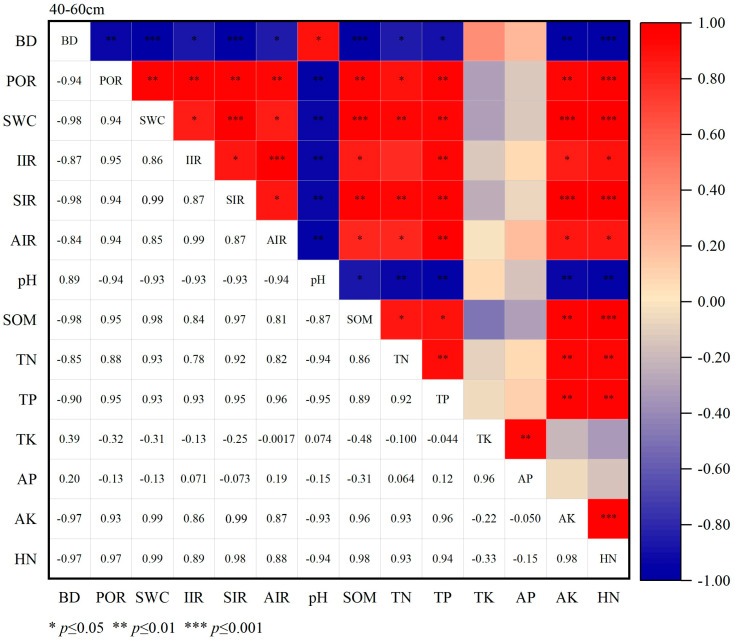
The correlation analysis among soil moisture, nutrients, and root traits across soil depths (40–60 cm).

**Figure 8:**
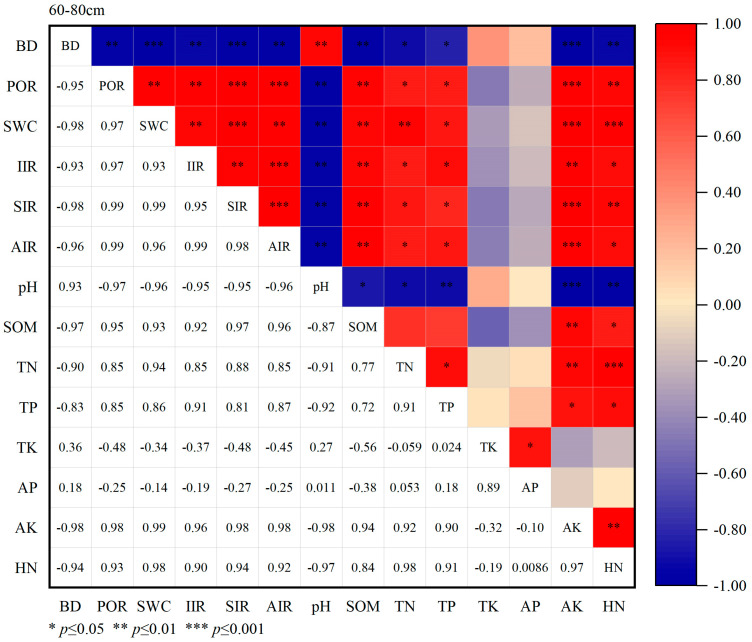
The correlation analysis among soil moisture, nutrients, and root traits across soil depths (60–80 cm).

**Figure 9:**
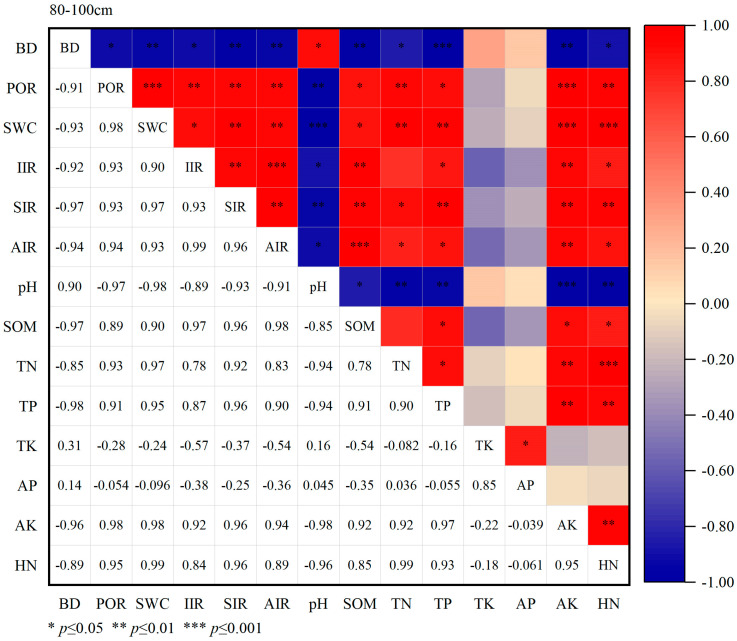
The correlation analysis among soil moisture, nutrients, and root traits across soil depths (80–100 cm).

Based on the correlation analysis among soil moisture, nutrients, and root traits across soil depths (0–100 cm), as presented in Figure 5, Figure 6, Figure 7, Figure 8 and Figure 9, the associations between soil moisture and nutrients in the Honghe Hani Rice Terraces exhibit pronounced vertical stratification. In the surface layer (0–20 cm), soil moisture showed a correlation coefficient of 0.92 with total porosity and correlation coefficients ranging from 0.89 to 0.99 with water infiltration rate, total nitrogen, total phosphorus, available potassium, available nitrogen, root number, and root weight density, all of which were statistically significant at the <0.01 level. In contrast, surface bulk density was not significantly correlated with available phosphorus or total potassium (r = 0.21–0.47) but exhibited a strong positive correlation with pH (r = 0.98) and significant to highly significant negative correlations with the remaining variables (r = −0.90 to −0.99). From 20 cm down to 100 cm, the correlation coefficients between soil moisture and all measured parameters gradually decreased, yet the overall direction and relative magnitude of the associations remained generally consistent with those observed in the surface layer. A notable spatial co-variation between surface soil moisture and root system size was detected; however, multiple competing explanations exist for this observed pattern. While increased root proliferation may enhance soil water retention through improved pore structure, concomitant variations in hydrothermal conditions along the elevational gradient could also drive synchronous changes in both root growth and soil moisture. Given that the present data were derived from spatial sampling, the direction of causality cannot be discerned, and thus the observed relationship should be interpreted as spatial co-variation rather than causal linkage. Elucidation of the underlying mechanisms will require further investigation through controlled water manipulation experiments or long-term in situ monitoring.

**Figure 10:**
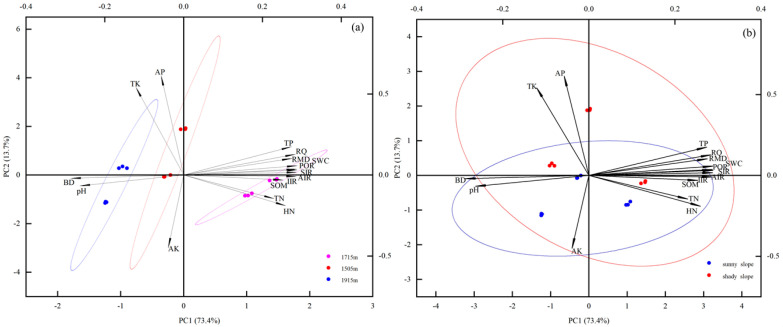
PCA plot of soil moisture, nutrients, and root parameters. (**a**) shows the effects of different elevations, and (**b**) shows the effects of different aspects.

The PCA results (Figure 10) show that PC1 accounts for 73.4% of the total variance. Parameters including TP, RQ, RMD, SWC, SIR, and POR exhibit high positive loadings on PC1, indicating that they serve as the primary environmental drivers. The clustering of soil physical properties such as soil water content and porosity reflects their collinearity and combined influence on water movement and retention. The association of SIR and IIR with soil respiration or microbial activity suggests that biogeochemical processes also play a significant role in environmental variability. In contrast, nutrient-related factors such as TN, HN, and AK show lower loadings on PC1, representing secondary influencing factors. SOM and IIR are distributed along the PC2 axis, implying their explanatory power in a secondary dimension. Additionally, elevation and aspect show significant correlations with PC1, demonstrating that altitudinal gradient and slope aspect indirectly regulate the spatial patterns of soil moisture, pore structure, and microbial activity by redistributing hydrothermal conditions.

Therefore, the ecological environmental variability in the Honghe Hani Terraces is primarily governed by soil physical properties and biogeochemical processes, with soil water content, porosity, and soil respiration being the main influencing factors. Elevation and aspect act as key topographic factors that indirectly shape the spatial distribution of these parameters through the redistribution of heat and moisture.

## 3. Discussion

This study aimed to investigate the altitudinal, aspect-related, and vertical differentiation patterns of soil physical properties and nutrient characteristics in the Yuanyang Hani rice terraces, as well as their synergistic relationships with root development. Due to experimental constraints, root indicators were limited to root number and root weight density, without including typical morphological parameters such as root length, root length density, and specific root length, which to some extent restricts an in-depth understanding of the interplay between root architecture and soil structure. Previous studies have demonstrated that fine roots (d ≤ 1 mm) contribute substantially to the formation of soil macropores through their root length density and root weight density, whereas coarse roots (d > 5 mm) show no significant correlation with this process [49]. In terrace ecosystems, roots primarily affect soil pore structure and water infiltration through the ≤5 mm diameter classes [50]. In the present study, root number and root weight density were significantly correlated with bulk density (r = −0.97 to −0.99), porosity (r ≥ 0.92), and infiltration rate (r ≥ 0.85). However, due to the absence of root length and diameter class distribution data, it was not possible to distinguish the differential contributions of various root diameter classes to structural amelioration, nor to evaluate the relationships between root physiological activity—as reflected by specific root length—and soil resource acquisition efficiency. This limitation implies that the conclusions drawn herein regarding the synergy between roots and soil physical properties remain at the biomass level, and the response mechanisms of root morphological plasticity to environmental gradients have not been fully elucidated.

With respect to the altitudinal gradient, soil bulk density, porosity, and nutrient contents (e.g., organic matter and total nitrogen) exhibited an optimal pattern at mid-elevations, which is largely consistent with the findings of Yao et al. on soil quality in the Hani terrace wetlands, who reported that soil organic matter, total nitrogen, total phosphorus, and comprehensive quality increased with elevation, except in the headwater areas [51]. The present study further reveals that this trend is not monotonically increasing; rather, the mid-elevation zone represents an optimum, whereas at high elevations, low temperatures suppress biological activity, leading to increased bulk density and reduced infiltration rates. This result is echoed by studies demonstrating significant aspect-related effects on soil physical properties, with higher soil particle fractal dimensions observed on north-facing slopes compared to south-facing slopes, reflecting the mediating role of slope aspect in regulating hydrothermal conditions and thus influencing the intensity of pedogenic processes [52]. Different slope positions systematically drive divergent patterns of carbon and nitrogen dynamics, as evidenced by studies on shifting cultivation systems, which have demonstrated that slope position exerts independent effects on the vertical distributions of both δ^13^C and δ^15^N. Notably, the enrichment of δ^15^N with increasing soil depth is particularly pronounced at the foot slope, reflecting the persistent modification of the nitrogen pool by erosion–deposition processes [53]. Previous research has also indicated that terracing practices significantly reduce soil water-stable aggregate content (from 61% to 24%) and final infiltration rates [52], which aligns with the abrupt increase in bulk density observed in the 20–40 cm soil layer due to tillage disturbance in this study, suggesting that the restructuring effects of anthropogenic cultivation on soil architecture are a common phenomenon across terrace systems in different regions. In summary, the altitudinal differentiation of soil properties in the Yuanyang Hani rice terraces is essentially the outcome of the tripartite interplay among hydrothermal conditions, biological activity, and anthropogenic tillage, with the mid-elevation optimum representing the most favorable trade-off among these three drivers. Nevertheless, owing to the lack of root morphological data, the interpretation of root–soil feedback mechanisms in this study remains incomplete. Future research should incorporate measurements of root length density and root diameter class distribution, combined with soil micromorphological analyses, to elucidate the differential contributions of distinct root diameter classes to pore structure and water transport dynamics.

## 4. Materials and Methods

### 4.1. General Situation of the Study Area

**Figure 11:**
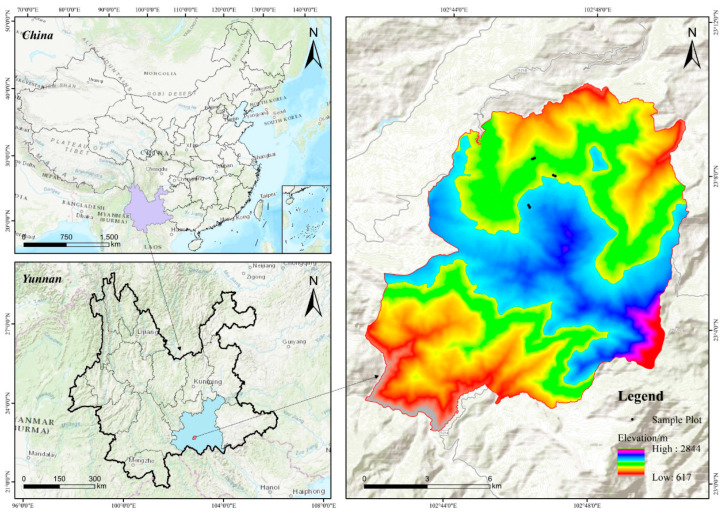
Geographical location map of the sampling sites in the study area.

In March 2025, six typical sampling plots were established in the Malizhai River watershed (102.77° E–102.78° E, 23.10° N–23.14° N), located near the core area of the Honghe Hani Rice Terraces in Yunnan Province, China. The elevation of the plots ranged from 1505 to 1915 m above sea level. The region experiences a subtropical mountain monsoon climate, with a mean annual temperature of 20.5 °C and annual precipitation between 1500 and 2000 mm, primarily concentrated from May to October. According to the World Reference Base for Soil Resources (WRB) classification system, the soils in the area are classified as *Haplic Arenosols (Transportic)*. The main soil layer is silty sandy loam, exhibiting a slightly dense to moderately dense structure, weak to moderate development, and a moderately moist state. The vegetation is characterized by subtropical evergreen coniferous and broad-leaved forests. The terraced fields support abundant herbaceous plants, with common species on the bunds including *Poaannua* L., *Cynodon dactylon*, *Alopecurus aequalis Sobol.*, *Polypogon fugax Nees ex Steud.*, *Pycreus flavidus (Retzius) T. Koyama*, and *Eleusine indica* (Figure 11).

### 4.2. Plot Establishment and Sampling

**Figure 12:**
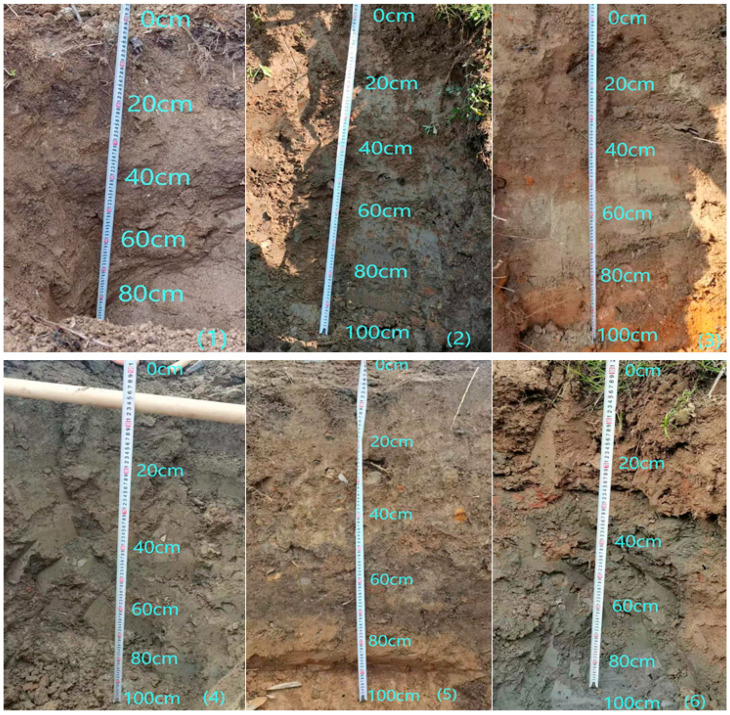
Geographical location map of the sampling sites in the study area. The figure displays six soil profiles collected along an elevation gradient under contrasting slope aspects: (**1**) 1505 m sunny slope; (**2**) 1505 m shady slope; (**3**) 1715 m sunny slope; (**4**) 1715 m shady slope; (**5**) 1915 m sunny slope; and (**6**) 1915 m shady slope.

**Figure 13:**
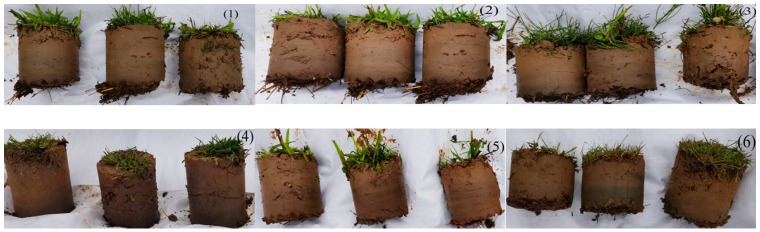
Photographs of root cores collected from different sample plots. Root core samples shown in the figure correspond to contrasting slope aspects along an elevational gradient: (**1**) 1505 m sunny slope; (**2**) 1505 m shady slope; (**3**) 1715 m sunny slope; (**4**) 1715 m shady slope; (**5**) 1915 m sunny slope; and (**6**) 1915 m shady slope.

This study selected three representative elevations along an altitudinal gradient—1505 m (low), 1715 m (middle), and 1915 m (high)—with three replicate plots established on both shady and sunny slopes at each elevation, resulting in a total of 18 quadrats of 10 m × 10 m. The elevation gradient was constrained by the fact that continuously distributed natural vegetation belts in this region are fully developed only within the aforementioned elevation range; beyond this interval, bedrock exposure increases substantially, making it difficult to ensure plot homogeneity and replicability. In addition, the herbaceous plant composition remains broadly similar across the three elevation bands, and the soil parent material consists uniformly of silty sand with a relatively homogeneous texture, thereby partly controlling the influence of underlying surface heterogeneity on the vertical variation of soil properties. Soil sampling followed a stratified design, with three sampling points arranged in an “S” shape within each quadrat, and samples were collected from five depth intervals: 0–20 cm, 20–40 cm, 40–60 cm, 60–80 cm, and 80–100 cm. The 0–100 cm profile was selected because it encompasses the primary rooting zone of herbaceous plants and is most directly influenced by near-surface atmospheric processes; below 100 cm, seasonal fluctuations in soil temperature, moisture, and organic matter inputs diminish markedly, and root biomass declines significantly, thereby limiting the indicator value for ecological processes. Undisturbed soil cores for physical property analyses were obtained by vertically pressing 100 cm^3^ stainless steel rings into each layer to determine bulk density and related parameters. Samples for chemical analysis and moisture content determination were collected using a soil auger, with no less than 1 kg per layer, and visible rock fragments and roots were manually removed prior to sealing and labeling in the field. Root samples were extracted using a root corer, and after washing, they were either refrigerated or oven-dried according to the specific analytical purposes. Fresh samples for chemical analysis were transported to the laboratory under refrigeration at 4 °C, air-dried, and passed through a 100-mesh sieve for storage; ring-core samples were transported with vibration protection to preserve structural integrity. Although this method provides a relatively detailed vertical resolution, the study is limited by the selection of only three elevations and two slope aspects, as well as the relatively small spacing among plots within each elevation band; consequently, the findings primarily reflect variation patterns along this specific topographic sequence (Figure 12 and Figure 13).

### 4.3. Analytical Methods

Soil bulk density and water content were determined using intact soil cores (100 cm^3^) collected synchronously at 20 cm intervals along the soil profile. Upon return to the laboratory, the exterior of each core ring was cleaned, and the fresh weight was recorded immediately. The samples were then oven-dried at 105 °C for 12 h to constant weight, with the endpoint defined as a mass difference of less than 0.01 g between two successive weighings. Soil porosity was calculated from bulk density and particle density, the latter being determined experimentally using the pycnometer method rather than adopting theoretical values.

Water infiltration parameters were obtained following the variable-head permeability test procedure (GB/T 50123-2019) [54], using a TST-55 permeameter (Nanjing Soil Instrument Factory Co., Ltd., Nanjing, China) equipped with a core ring of 4.0 cm height and 6.18 cm internal diameter. Soil samples were packed flush with the ring edges to minimize compaction and structural disturbance. The test water temperature was recorded to the nearest 0.5 °C, and the measured hydraulic conductivity values were corrected to the standard temperature of 20 °C. Each soil sample was assayed in triplicate; if the relative deviation among replicates exceeded 5%, the measurement was repeated.

For chemical property analyses, soil pH was measured in a 1:2.5 (*w*/*v*) soil–water suspension using a glass electrode immersed for 30 min to allow stabilization. Before each batch of measurements, the electrode system was calibrated with pH 4.01 and 6.87 standard buffer solutions. Soil organic matter was determined by the external heating method, with the oil bath maintained at 185 ± 2 °C and heating time controlled to within 5 min. The titration endpoint was uniformly defined as the appearance of a bright green color upon a slight excess of 0.2 mol/L ferrous ammonium sulfate. Each batch included one blank and one national primary reference material (GSS series) for quality control. Total nitrogen was measured by semi-micro Kjeldahl digestion with a fixed distillation time of 5 min, and the titration endpoint for the receiving solution was determined as a stable purplish-red color. Total phosphorus was determined by the acid digestion–molybdenum antimony anti-spectrophotometric method, with color development carried out at a constant temperature of 25 °C for 30 min. Available phosphorus was extracted using the Olsen method (0.5 mol L^−1^ NaHCO_3_, pH 8.5), with shaking for 30 min under temperature control within ±2 °C. Total potassium and available potassium were both determined by flame photometry; available potassium was extracted with 1 mol L^−1^ ammonium acetate at a constant temperature of 25 °C, and after shaking for 30 min, the extract was filtered immediately to avoid adsorption-induced changes. Hydrolysable nitrogen was determined by the alkaline diffusion method: boric acid indicator solution in the inner chamber of the diffusion dish was sealed promptly after adding alkali to the outer chamber, and diffusion was allowed to proceed at 40 °C for 24 h, followed by titration with standard acid to a faint red endpoint.

Root sampling was conducted using a layer-by-layer excavation method, with 0.5 m × 0.5 m × 0.2 m soil blocks collected from each depth interval. The blocks were placed in gauze bags and soaked in water for 12 h, after which roots were washed under running water through a 0.5 mm sieve until no visible soil particles remained. Live roots were identified based on color (light yellow or brown), flexibility, and cortical integrity; dark gray roots that were brittle and broke upon touching were classified as dead and excluded. After washing, root samples were blotted dry with absorbent paper to remove surface moisture and weighed for fresh mass within 1 h. They were then oven-dried at 65 °C for 24 h, followed by further drying at 80 °C to constant weight (defined as a weight difference of <0.5 mg between successive weighings), and finally weighed after cooling to room temperature. The fresh-to-dry mass ratio was recorded for each sample to facilitate outlier identification. All weighing procedures were performed using an analytical balance with 0.1 mg precision. In each batch, 20% of the samples were randomly selected for duplicate analysis to verify consistency in sample preparation and weighing stability.

### 4.4. Data Processing

Data were initially processed and summarized using descriptive statistics in Excel 2020. Statistical analyses, including Pearson correlation analysis, Kaiser–Meyer-Olkin (KMO) measure, and Bartlett’s test of sphericity, were performed using SPSS 19.0 to assess data suitability. Key drivers of variation within the soil system were identified through principal component analysis (PCA). All figures were generated using Origin 2021, with data presented as mean ± standard deviation.

## 5. Conclusions

This study systematically demonstrates that the physical and chemical properties of the 0–100 cm soil layer in the Yuanyang Hani terraced fields exhibit significant differentiation with respect to altitude, slope aspect, and soil depth. The results indicate that the mid-altitude zone represents the optimal structural belt, characterized by the lowest bulk density (1.10–1.17 g cm^−3^), total porosity exceeding 54%, and steady-state infiltration rates of 1.31–1.34 mm min^−1^, accompanied by the highest contents of organic matter, total nitrogen, and available nutrients across all altitudes. In contrast, the low-altitude zone shows relatively high bulk density due to tillage-induced compaction, while the high-altitude zone exhibits denser soil structure resulting from low-temperature physical weathering and the lowest infiltration capacity (0.93–0.96 mm min^−1^). At the same altitude, the bulk density on north-facing slopes is 0.05–0.08 g cm^−3^ lower than that on south-facing slopes, whereas soil water content and infiltration rate are 2–5% and 4–8% higher, respectively, with the aspect effect being most pronounced at mid-altitudes. Vertically, the 0–20 cm plough layer is loose, whereas the 20–40 cm layer exhibits an anomalous phenomenon characterized by a sharp increase in bulk density accompanied by concurrent increases in porosity and infiltration rate, which is attributed to the coexistence of mechanically compacted pore redistribution, micropores, and biopores. Below 40 cm depth, the soil is dominated by matrix pores, resulting in slow water movement. Root number and root weight density are significantly negatively correlated with bulk density (r ≤ −0.97) and significantly positively correlated with water content, porosity, hydrolyzable nitrogen, and available potassium (r ≥ 0.96). However, since the root indicators only include number and biomass without distinguishing root diameter classes, the current conclusions remain at the level of biomass synergy, and the contribution pathway of root morphological plasticity to pore structure differentiation cannot yet be determined. Soil pH is significantly negatively correlated with root traits (r ≤ −0.93), suggesting that acidic stress may constrain root proliferation.

## Data Availability

The original contributions presented in this study are included in the article.

## Abbreviations

The following abbreviations are used in this manuscript:

AIR	Average Infiltration Rate
BD	Bulk Density
IIR	Initial Infiltration Rate
POR	Porosity
SIR	Stable Infiltration Rate
SWC	Soil Water Content
TN	Total Nitrogen
TP	Total Phosphorus
TK	Total Potassium
HN	Available Nitrogen
AP	Available Phosphorus
AK	Available Potassium
RQ	Root Quantity
RMD	Root Mass Density
